# Dual regulation of autophagy: paradoxical effects on tumor angiogenesis

**DOI:** 10.3389/fcell.2026.1872152

**Published:** 2026-06-29

**Authors:** Shanshan Xiang, Xue Gong, Jiangyuan Zhou, Tong Qiu, Yuru Lan, Zixin Zhang, Yi Ji

**Affiliations:** Division of Oncology, Department of Pediatric Surgery, West China Hospital of Sichuan University, Chengdu, China

**Keywords:** activator, angiogenesis, autophagy, dual regulation, inhibitor, tumor

## Abstract

Autophagy plays a critical role in dynamic cell growth under different environmental conditions and maintains cellular homeostasis and cell viability in response to different cellular injuries, especially in tumor angiogenesis, where there is a controversial dual role. Angiogenesis plays a key role in facilitating tumor development. Therefore, investigating the mechanism of action of autophagy in the flexible shift of tumor angiogenesis is essential for antiangiogenic clinical therapy, and modulating the autophagy process is expected to constitute a new strategy to inhibit tumor angiogenesis and stop tumor growth and spread. This review highlights the close connection between autophagy and tumor angiogenesis. We also attempt to explain the seemingly contradictory promotion or inhibition of angiogenesis in tumor development reported in past studies. We focus on the potential clinical value of autophagy agonists and inhibitors in antiangiogenic therapy, which will provide new theoretical bases for antitumor therapy and may lead to innovative therapeutics.

## 1 Introduction

Autophagy is a key homeostatic pathway that promotes the degradation and recycling of cellular material, a process that maintains metabolic homeostasis and other critical processes within eukaryotic cells (Galluzzi et al., 2017). Current nonselective types of autophagy are categorized into macroautophagy, microautophagy and molecular chaperone-mediated autophagy (Hassanpour et al., 2018). Autophagy is not only a stress response to perturbations in the homeostasis of the internal environment but also more likely to be induced by cellular stressors, including hypoxia and reactive oxygen species (Kroemer et al., 2010). The core function of these signals is to balance metabolic homeostasis to support the cellular requirements for life activities (Altman and Rathmell, 2012), and disorders in the autophagic response resulting from excessive or reduced autophagy may underpin the development of cardiovascular diseases (Lavandero et al., 2015), metabolic diseases (Wang et al., 2020), tumors (Rubinsztein et al., 2012) and other diseases. Recent studies have revealed that the bidirectional regulation of autophagy enables endothelial cells to adapt dynamically to tumor microenvironments such as hypoxia and effectively regulate pathological angiogenesis, which is one of the hallmarks of solid tumors (Schaaf et al., 2019). Tumor development relies on the initiation of tumor angiogenesis (Sherwood et al., 1971), which provides oxygen and nutrients to supply tumor cells for survival. A previous clinical trial demonstrated that the coadministration of antibodies targeting VEGF effectively prolonged the survival of patients with metastatic colorectal disease, providing proof-of-concept that antiangiogenic therapies can be used to successfully treat cancer (Hurwitz et al., 2004). As a new target for antiangiogenic therapy, autophagy has been shown in some studies to potentially promote angiogenesis (Tao et al., 2023). In this review, we focus on contradictory analyses of conflicting data from different studies to discuss in detail the mechanisms by which autophagy occurs.

## 2 Physiological functions of autophagy

Autophagy promptly removes damaged or dysfunctional organelles to prevent the accumulation of aberrant aggregates (Yamashita and Kanki, 2017) and limits deoxyribonucleic acid damage-induced instability; the ultimate goal is to reduce metabolic stress (e.g., nutrient deprivation, growth factor depletion, and hypoxia) (Levine and Kroemer, 2008). When autophagy becomes dysfunctional, cells release proapoptotic factors (e.g., cytochrome c and apoptosis-inducing factors) to activate apoptotic pathways (Quarato et al., 2023). Moreover, endothelial cells are in direct contact with the blood environment and are susceptible to direct damage from oxidative stress and inflammation. Autophagy can help reduce the level of oxidative stress and inflammation in endothelial cells and may affect the secretion of hormones by endothelial cells, which may influence functions such as angiogenesis and thrombosis and play important roles in maintaining the functional stability of endothelial cells.

## 3 Regulation of tumor angiogenesis

### 3.1 Basic processes of angiogenesis

Angiogenesis involves the formation of new blood vessels from existing vessels. The entire process includes degradation of the basement membrane; activation, proliferation and migration of endothelial cells; and neointima formation (Fan et al., 1995). The initial formation of the vascular system is followed by remodeling and optimization to subsequently form arteries, veins and capillaries. In contrast, there are different forms of tumor angiogenesis, including bone marrow-derived angiogenesis (bone marrow-derived endothelial progenitor cells differentiate into endothelial cells to form blood vessels), angiogenic mimicry (tumor cells form vascular channels), vascular coselection (tumor cells invade normal tissues to utilize the existing circulatory system to collect nutrients and oxygen), and angiogenesis of cancer stem cell origin (cancer stem cells differentiate into endothelial cells) (Zhang et al., 2024). Angiogenesis often manifests as the transition of endothelial cells from a quiescent state to a highly active, proliferative, and migratory state (Eelen et al., 2018), a process that requires a large amount of energy to support cell proliferation and migration, thus ensuring the normal progression of processes such as vessel sprouting, especially glucose metabolism, which is thought to be an important driver of angiogenesis (Yang et al., 2023). Angiogenesis is carried out in an orderly manner under the coordination of multiple signaling pathways, and the breakdown of the coordination between the pathways results in abnormal vascular processes, which lead to the occurrence of processes such as tumors.

### 3.2 Relationship between angiogenesis and tumor development

**FIGURE 1:**
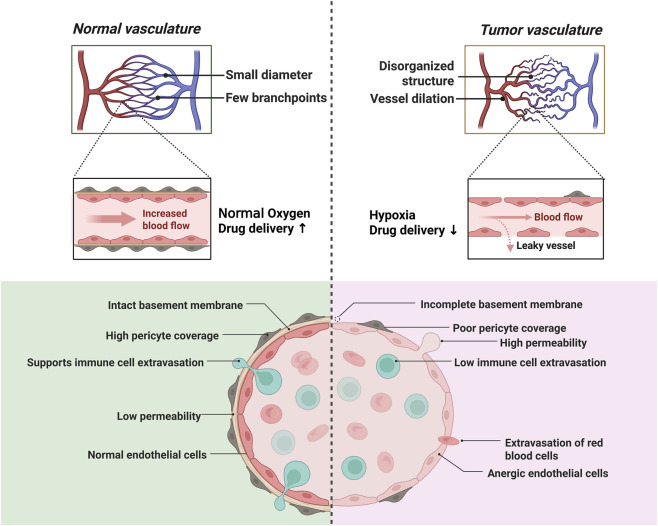
Comparison of the morphology and function of tumor and normal blood vessels. (1) The normal vasculature consists of arterioles, venules, and capillaries that exhibit an organized, hierarchical branching pattern. Endothelial cells are supported by the basement membrane and pericyte coverage and are tightly connected by stable cell‒cell junctions. (2) The tumor vasculature is characterized by an unorganized vascular network that lacks distinct stratification because of the sustained and unbalanced expression of angiogenesis-promoting and angiogenesis-inhibiting factors. This process is characterized by decreased blood flow, endothelial cell germination, endothelial cell junction disruption, pericyte coverage loss, and increased blood leakage, leading to tissue hypoxia. In addition, tumor endothelial cells have abnormal basement membranes, which are loosely bound to endothelial cells and are variable in thickness.

Angiogenesis occurs primarily during embryonic and fetal development, whereas in adults, angiogenesis generally occurs only in the placenta during cyclic ovarian and gestational periods (Li and Ding, 2021). However, in the presence of estrogen, hypoxia, inflammation, and other disturbances (Xiang et al., 2024), endothelial cells maintain high plasticity to sense and respond to angiogenic signals to limit the disease process (Jeong et al., 2020). Once angiogenesis is dysregulated, there is a high probability that it will further support disease progression. The hypothesis that angiogenesis and tumor growth may be interdependent was first proposed by Folkman et al., in 1971 (Folkman, 1971) and was subsequently confirmed by evidence from numerous studies, including the discovery of pro- and antiangiogenic factors (Masiero et al., 2013). The dynamic balance between pro- and antiangiogenic factors in the tumor microenvironment can be disrupted (Hanahan and Folkman, 1996). Vascular endothelial growth factor (VEGF)/vascular endothelial growth factor receptor-2 signaling may constitute an autocrine cycle (Ji et al., 2014); such conditions result in the transformation of the quiescent vasculature system into a proliferative vasculature system to satisfy the metabolic demands of the tumor for oxygen and nutrients. High levels of angiogenesis play crucial roles in tumor growth, tumor invasion and poor prognosis (Li B. et al., 2024; Kong et al., 2025). The tumor vasculature generally consists of tumor endothelial cells, tumor mural cells (pericytes and smooth muscle cells), and the basement membrane (Baluk et al., 2005). Pericytes can be regulated by the Jagged1/Notch3 signaling pathway to adapt to vascular growth (Ji et al., 2016). The tumor vasculature is heterogeneous across different tumor types and subtypes, with large differences in morphology, function and gene expression patterns (Maishi et al., 2019). Owing to the presence of persistent proangiogenic signals in tumors, newly formed vascular networks fail to undergo pruning and maturation (Baluk et al., 2005). The tumor vasculature is morphologically characterized by excessive branches and blind ends, discontinuous endothelial cell junctions, and defective basement membranes and pericyte cover (De Palma et al., 2017); this leads to increased vascular permeability, erythrocyte infiltration into the extravascular space, an increased risk of hemorrhage, and impaired drug delivery to the lesion site (Hashizume et al., 2000) (Figure 1). These morphologies also lead to uneven blood flow within the tumor parenchyma, triggering dynamic areas of persistent or intermittent hypoxia (Bennewith and Durand, 2004). This variable oxygenation state affects tumor sensitivity to radiotherapy and chemotherapy (Durand, 2002). Quantifying this transient hypoxia in solid tumors and exploring the impact of indirect hypoxia on clinical prognosis is expected to influence individualized clinical treatment strategies, including the timing and dosing of chemotherapy.

## 4 Bidirectional roles of autophagy in tumor angiogenesis

It is now widely accepted that the role of autophagy in tumors depends on the tumor type and microenvironment. In ovarian cancer, triple-negative breast cancer, and prostate cancer (Gwin et al., 2013), induced autophagy primarily protects cells rather than inducing apoptosis. It mainly counteracts drug-induced changes in the tumor microenvironment to maintain tumor drug resistance; therefore, inhibiting autophagy may be an effective strategy for treating these types of tumors. However, in certain tumor types, autophagy can also inhibit tumor progression. High expression of the autophagy protein beclin-1 in uveal melanoma is strongly associated with a favorable prognosis (Broggi et al., 2020), potentially exerting a significant influence on tumor angiogenesis and thereby inhibiting tumor development. While prophase autophagy inhibits tumorigenesis by reducing oxidative stress and eliminating genomic instability (Mathew et al., 2007), developing tumors utilize autophagy to cope with microenvironmental pressure, such as metabolic and hypoxic stress conditions (Katheder et al., 2017). In addition, oxidative stress due to insufficient energy supply and demand in some tumor cells is transmitted to surrounding tumor stromal cells, and the effect of autophagy on the crosstalk between pericytes and other cells is important for vascular homeostasis. Most evidence suggests that autophagy promotes tumor angiogenesis, but controversial evidence has shown that cell death can be attributed to unrestricted levels of autophagy rather than to caspase-dependent cell death (Reef et al., 2006). A more plausible explanation is that the catabolism of autophagy primarily maintains cell survival. However, this autophagic depletion ultimately leads to a critical threshold of intracellular material digestion, the direct activation of prodeath molecules, or the degradation of specific molecules or organelles necessary for cell survival (Liao et al., 2019). This ultimately triggers type II nonapoptotic programmed cell death. However, substantial experimental evidence supporting this speculation is currently lacking. Autophagy plays a dual role in tumors, and whether it directly leads to death or mediates the death process is still controversial. Before the paradoxical functions of autophagy are understood, it is generally believed that autophagy initially acts as a cellular protective mechanism, whereas excessive autophagy promotes cell death (Table 1). This process is typically dynamically regulated by the magnitude of cellular stress and disease progression. When the cumulative damage caused by internal and external stimuli exceeds the lysosomal clearance capacity, protective autophagy transitions to lethal autophagy; that is, this tipping point defines the autophagy flux threshold. Before quantifying this threshold, we need to quantify autophagic flux. The initial approach involves measuring the expression levels of key autophagy proteins (including LC3B and p62) in the presence of stressors or inducers and quantifying the number of autophagosomes, which has become the gold standard for assessing autophagy activity. Additionally, live-cell imaging provides supplementary observations of the dynamic process of autophagosome degradation. The autophagy flux threshold essentially defines the boundary between pure autophagy and cell death (including autophagic cell death and other death modes such as apoptosis) and can be quantitatively attributed to differences in the rate of lysosomal degradation of autophagic substrates. Given that, under steady-state conditions of the autophagy system, the rates of each step in the autophagy pathway are equal, it becomes possible to experimentally quantify autophagosome flux by inhibiting a specific step. Nonlethal and lethal autophagic flux can be predicted using parameters such as the rate of autophagosome degradation (Loos et al., 2014) and the dynamics of autophagosome vesicle transport (Martin et al., 2013). Assessment of the existence of an autophagy flux threshold through phenotypic observations—i.e., when adaptive autophagy activity exceeds that required to promote autophagy—reveals morphological features of autophagy-mediated cell death, including enhanced cell–substrate adhesion, endoplasmic reticulum (ER) expansion and fragmentation (early stage), and ER depletion and disappearance (late stage), as well as nuclear envelope invagination (early stage) and localized swelling of the perinuclear space (late stage) (Liu and Levine, 2015), as well as a unique dependence on the sodium-potassium ATPase (Liu et al., 2013). Given the critical role of the endoplasmic reticulum in autophagosome biogenesis (Wu et al., 2026), under conditions of extremely high autophagic flux, the endoplasmic reticulum may be depleted as a source of autophagosomes; therefore, endoplasmic reticulum depletion may also be important.

**TABLE 1:** Differences between protective autophagy and lethal autophagy.

Type	Trigger	Autophagy flux intensity	Key mediating molecules	Cellular outcome
Protective autophagy	mild stress	low	mTOR, p62, LC3	survival, metabolic adaptation
Lethal autophagy	severe/prolonged stress	persistently elevated	Beclin 1, caspase	apoptosis, nonapoptotic cell death

### 4.1 Autophagy promotes tumor angiogenesis

Autophagy-deficient mice show signs of energy depletion and die of a lack of nutrition after 1 day of life, demonstrating that autophagy can be used as a survival mechanism to replenish energy metabolites and maintain viability (Kuma et al., 2004). Autophagy induced *in vitro* under hypoxic (Li et al., 2016), hyperglycemic (Du et al., 2017) and thermal denaturation (Liang et al., 2018) environments effectively increases cell migration and tube formation and is involved in retinal neovascularization. Additionally, in the context of pathological hypoxia/reoxygenation-associated neovascularization, endothelium-specific ATG5 defects in a model of retinopathy of prematurity affect only pathological angiogenesis (Sprott et al., 2019). Under stress conditions, autophagy activation effectively promotes the formation of angiogenic mimics that provide nutrients, oxygen, and invasive pathways to tumors, maintaining the survival and invasive capacity of tumor cells under stress (Ding et al., 2014). Autophagy stimulated by rapamycin maintains the viability of human umbilical vein endothelial cells (HUVECs) exposed to high concentrations of glucose, affects cell migration and renal tubulogenesis *in vitro*, and thus increases the angiogenic potential of endothelial cells (Rezabakhsh et al., 2017). Autophagy is required for endothelial cell proliferation, migration, and angiogenesis mediated by proangiogenic growth factor (angiogenic factor with G-patch and FHA domain 1), which can help to effectively enhance therapeutic neovascularization (Lu et al., 2016). In osteoporosis studies, arginine was shown to promote angiogenesis by promoting PINK1/Parkin- and BNIP3-mediated mitochondrial autophagy (Shen et al., 2024). These potential drugs can mediate angiogenesis through the autophagy-induced upregulation of the expression of angiogenic factors (peroxisome proliferator-activated receptor β/δ (Pan et al., 2023) and transforming growth factor-β1) (Li et al., 2022). In most cases, the increase in autophagy acts as a cytoprotective response to inhibit antitumor drug-induced apoptosis and antiangiogenesis and maintains or promotes angiogenesis (Meng et al., 2019), resulting in poor antiangiogenic effects. As an autophagosome marker, LC3B shows moderate to strong staining in most tumors; high LC3B expression is closely associated with the proliferation, invasion, and metastasis of malignant tumors (Lazova et al., 2012), supporting the notion that advanced human tumors typically exhibit increased autophagic flux. Most evidence from these studies suggests that autophagy promotes angiogenesis in tumor diseases. The role of autophagy in promoting angiogenesis is related, on the one hand, to the increased production and release of vascular growth factors and promigratory cytokines and, on the other hand, to the dynamic adaptation of vascular endothelial cells to intracellular and extracellular stress environments under hypoxic conditions within tumors, thereby maintaining biological functions such as proliferation and migration and promoting tumor progression.

### 4.2 Autophagy inhibits tumor angiogenesis

Depending on the cellular environment, stress signaling-induced increases in autophagy may generate energy and other substances through the degradation of intracellular material as a survival mechanism. However, the excessive degradation of cellular contents due to excessive autophagy may also be a mechanism of death, which in turn affects angiogenesis. The role of autophagy in cell death can be categorized into autophagy-dependent cell death (autophagic cell death) and autophagy-mediated cell death (Galluzzi et al., 2018), which serve as the basis for the initiation of other modes of death (Liu et al., 2023). In the former group, only elevated levels of autophagy were observed in the dead cells, with no signs of apoptosis or other forms of cell death, indicating that cell death was directly caused by autophagy. In the latter group, increased levels of autophagy mediated forms of cell death, such as apoptosis. A review of the different roles of autophagy in cell death has been conducted, and the major autophagy-associated cell death modes have been comprehensively reviewed (Liu et al., 2023). Evidence for the tumor-suppressive role of autophagy primarily stems from studies on Beclin-1, a Bcl-2-interacting protein. Heterozygosity for Beclin-1 increases the likelihood of spontaneous malignancy and has been identified as a dose-dependent tumor suppressor gene (Yue et al., 2003; Qu et al., 2003). Furthermore, by establishing a link between Beclin-1 and the Akt pathway (Wang et al., 2012), it has been determined that the tumor-suppressive activity of Beclin-1 stems from its characteristic autophagic function. Akt signaling plays a crucial role in angiogenesis, and its effects on cell growth, proliferation, metabolism, and vascular biology have been summarized in recent reviews (Manning and Cantley, 2007; Manning and Toker, 2017). Additionally, direct evidence supports the tumor-suppressive role of autophagy, including a significantly increased incidence of sarcomas (Mariño et al., 2007) and hepatic adenomas (Takamura et al., 2011) in mice deficient in Atg4c, Atg5, and Atg7. The effects of autophagy on tumors are influenced by various factors, including tissue type, tumor microenvironment, and tumor subtype. A p62-dependent response to counteract oxidative stress has been reported in melanoma cells exposed to ultraviolet radiation (Sample et al., 2018). Ceramide interacts with LC3B-II to target lethal mitochondrial autophagy and trigger caspase-independent cell death (Sentelle et al., 2012). Interfering with the autophagic response by knocking down Beclin 1 expression significantly increases endothelial apoptosis, inhibits cell proliferation, and effectively inhibits angiogenesis (Ramakrishnan et al., 2007). These results are expected to lead to the use of autophagy as a new target to enhance the therapeutic effect of angiogenesis inhibitors. The inhibitory effect of autophagy on angiogenesis is manifested primarily through its targeting of endothelial cell death. Excessive autophagy can induce various forms of cell death, such as autophagic cell death and apoptosis, thereby inhibiting tumor angiogenesis. Currently, most studies on whether autophagy inhibits or promotes angiogenesis have not directly incorporated the threshold of autophagic flux into their analytical frameworks; this represents a critical area that requires urgent attention in future research. Existing studies have largely focused on the direct effects of autophagy induced by single concentrations of reagents on the biological functions of vascular endothelial cells; consequently, the experimental results from multiple studies may appear somewhat contradictory at the superficial level. Exploring the role of autophagic flux thresholds in autophagy-mediated cell death pathways and elucidating their functions in other cell death pathways requires a deep understanding of the dynamic and systemic relationship between autophagic flux and cell death.

### 4.3 Crossregulation of tumor angiogenesis by the autophagy signaling pathway

**FIGURE 2:**
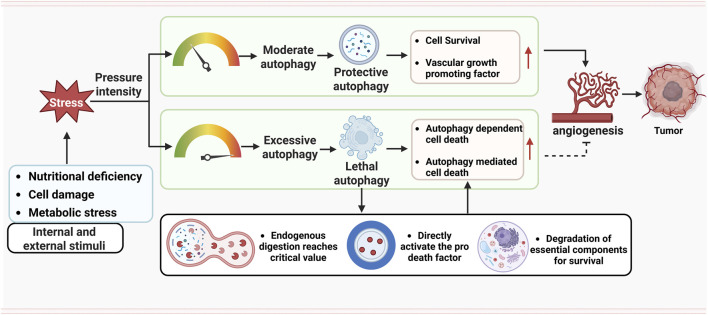
Bidirectional regulatory mechanisms of autophagy in tumor angiogenesis. Under stress conditions, autophagy has a bidirectional effect: moderate autophagy maintains cellular homeostasis and survival, whereas excessive autophagy induces cell death through mechanisms such as reaching a threshold of endogenous digestion, direct activation of pro-apoptotic factors, or degradation of essential survival components. Autophagy bidirectionally regulates tumor angiogenesis: it can promote angiogenesis to support tumor development or inhibit angiogenesis under specific conditions, thereby exerting an antitumor effect.

**FIGURE 3:**
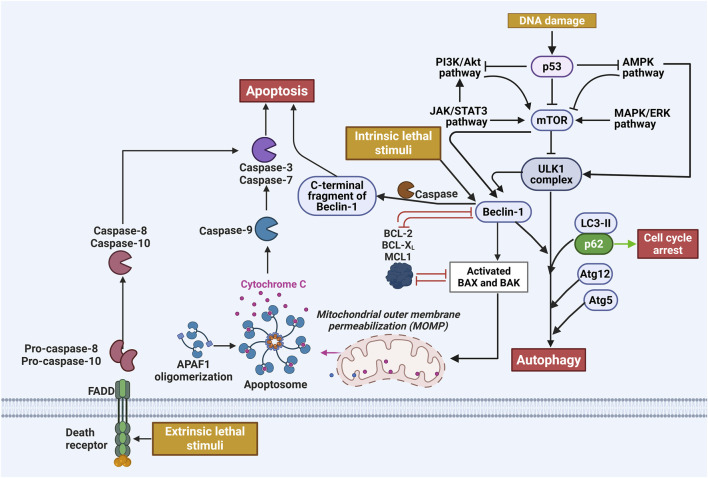
Balancing role of autophagy and apoptosis signaling pathways in determining cell fate. When cells face external and internal stressors, caspase protein family members and P53-related signaling pathways are activated, together regulating the dynamic balance between autophagy and apoptosis. In a given scenario, when the intensity of autophagy signaling exceeds that of apoptosis, cells tend to choose survival strategies to maintain their vital activities. However, if cells are exposed to a continuously stressful environment for a long period, the intracellular balance gradually shifts, prompting the cells to shift from survival to apoptosis, which ultimately leads to cell death.

Although most of the evidence suggests that autophagy promotes cell survival, paradoxically, extensive autophagy is usually observed in dead cells. The relationship between autophagy and cell death is more clearly understood, with a large amount of crosstalk between them that collectively influences angiogenesis either in a promoting or inhibitory manner (Figure 2). The autophagic flux threshold indicates the transition of a cell from a potentially reparable stress state to a mature death state; this process is not a linear transition but involves specific kinetic switches in autophagic flux, highlighting signaling crosstalk among signaling molecules such as mTORC1, p53, p62, and AMPK, as well as organelle-specific autophagy (Bahar et al., 2026) (Figure 3). The most characteristic feature distinguishing autophagy from other modes of cell death is the interaction between the BH3 domain of the autophagic protein Beclin-1 and the antiapoptotic protein Bcl-2. Beclin-1 is the mammalian homolog of Atg6 and plays a crucial role in the cellular biological functions of autophagy and apoptosis; the link between Beclin-1 and the Akt signaling pathway underscores the importance of autophagy in tumor progression (Wang et al., 2012). Under nutrient-rich conditions, Beclin-1 binds to Bcl-2, and the autophagic activity of Beclin-1 is inhibited by the localization of Bcl-2 to the endoplasmic reticulum (Liang et al., 1998). The binding and dissociation of Beclin-1 and Bcl-2 are positively and negatively regulated by various intracellular mechanisms (Maejima et al., 2013; Zalckvar et al., 2009) to inhibit or promote autophagy. Additionally, some autophagy-related proteins can act as pro-apoptotic factors to initiate apoptosis. The apoptotic mechanism can inhibit autophagy by triggering caspase-mediated cleavage of Beclin-1; the resulting C-terminal fragment of Beclin-1 is localized to the mitochondria, where it induces the release of proapoptotic factors and enhances apoptosis (Wirawan et al., 2010). The inhibition of Caspase-10 leads to uncontrolled, high-intensity autophagy, resulting in cell death that lacks the hallmarks of apoptosis (Lamy et al., 2013). The regulation between caspases and Beclin-1 supports a mathematical model of autophagy-mediated cell fate decisions to simulate the effects of increased stress levels on autophagy and apoptosis signaling (Wirawan et al., 2010; Tavassoly et al., 2015); the translational process of this model can help support autophagy-stage-dependent therapeutic strategies. Among the inhibitory factors in autophagy, mTORC1 is the most well defined (Senapati et al., 2025), with AMPK acting as its upstream inhibitory signal (Chun and Kim, 2021)^.^ AMPK can also directly stimulate autophagy by phosphorylating ULK1 (Egan et al., 2011) and Beclin-1 (Medeiros et al., 2020)^,^ whereas mTOR inhibits autophagy by phosphorylating and inactivating key regulators, including ULK1 (Li et al., 2025), VPS34, and Beclin-1 (Ding et al., 2022). p53, a well-studied tumor suppressor gene, can activate AMPK via noncanonical pathways to modulate autophagy (Wang et al., 2023). As a major regulator of autophagy, p62 interacts with LC3 to facilitate the transport of autophagosomes to lysosomes for degradation. Additionally, p62 interacts with caspase-8 to trigger apoptosis (Jung and Oh, 2019). Nip3-like protein X was initially described as a proapoptotic factor or a cell death factor (Chen et al., 1999); BNIP3 also participates as a proapoptotic protein in hypoxia-induced mitochondrial autophagy (Quinsay et al., 2010). Beclin-1 and the Bcl2 complex, which are key regulators of autophagy, inhibit autophagy, but when apoptosis is inhibited, high levels of autophagy can serve as a cell death effector mechanism (Madeo et al., 2010). Downregulated Beclin-1 expression promotes STAT3 phosphorylation in colorectal cancer cells, and activation of the JAK/STAT3 axis promotes colorectal cancer metastasis (Hu et al., 2020). In addition, in lung adenocarcinoma cells, activated and enhanced JAK/STAT3 increases VEGF secretion in the tumor microenvironment and promotes tumor angiogenesis (Xia et al., 2022). The PI3K/Akt/mTOR pathway also plays an important role in tumor angiogenesis, and astragaloside A can activate this pathway by interfering with human umbilical vein endothelial cells, thereby increasing the level of VEGF expression (Cheng et al., 2019). The target protein of rapamycin, mTOR, is among the most studied proteins in the regulation of autophagy. Although it is possible that in autophagy, which has dual effects, protective and lethal autophagy are triggered simultaneously, at a certain disease stage, one of these types of autophagy will dominate (Fu et al., 2016). Once the autophagy threshold is reached, cellular damage signals are stabilized, establishing the necessary conditions for irreversible cell death; the resulting cellular imbalance then translates into a sustained positive feedback loop.

## 5 Autophagy-targeted therapy for tumor angiogenesis

### 5.1 Potential of autophagy activators in tumor angiogenesis therapy

However, when tumor therapy is considered from another perspective, it is possible to promote the occurrence of apoptosis by promoting excessive autophagy (lethal autophagy) in tumor cells, thus enhancing antitumor effects. Some studies have shown that ciclopirox platinum (IV) synergistically activates intracellular apoptotic pathways by promoting mitochondrial autophagy in tumor cells and inhibiting tumor angiogenesis, thereby inhibiting tumor cell metastasis (Li S. et al., 2024). In the treatment of osteosarcoma, tretinoin induces significant cellular autophagy, thereby inhibiting angiogenesis and inducing apoptosis in osteosarcoma in a dose-dependent manner (Li et al., 2018). In addition, Chonglou saponin II induced autophagy in endothelial cells by modulating the PI3K/AKT/mTOR signaling pathway, thereby affecting angiogenesis and inhibiting the proliferation, migration and invasion of cervical cancer cells (Cheng et al., 2023). Excessive autophagy similarly inhibits the proangiogenic effects of monoflavin on bone marrow-derived endothelial progenitor cells (Wang et al., 2018). Although these studies did not report high or low levels of autophagy, these drugs generally promote tumor cell death by increasing the levels of lethal forms of autophagy. Given that most current autophagy-inducing drugs broadly stimulate autophagy, the pathways upstream of autophagy are equally involved in many physiological activities other than autophagy (Moors et al., 2017). SQSTM1-mediated mitophagy inducers are thought to increase endogenous levels of mitochondrial autophagy, exposing only organelles in need of recycling without compromising the bioenergetics of the entire cellular network (East et al., 2014), and may be promising chemical drug candidates. Since lethal autophagy is rarely triggered in cells under physiological or pathological conditions, precisely targeting the downstream components of autophagy may be an effective strategy for the development of autophagy-selective activators for clinical translational therapeutics, providing new avenues for future drug development. In addition to the effects of monophasic drugs, the promotion of autophagy by sorafenib is bidirectional, and the activation of autophagy inhibits apoptosis, mediates cellular chemoresistance in hepatocellular carcinoma cells (Lin et al., 2020), and induces autophagy-dependent iron death in hepatocellular carcinoma cells (Liu et al., 2021). Thus, autophagy may be involved in different processes in the same cell, but specific and precisely targeted autophagy inhibitors and activators are lacking in the clinic. Under conditions of cytotoxic stress, increased autophagy is typically associated with chemoresistance. The autophagic flux observed under these conditions is significantly greater than baseline levels but has not yet reached lethal levels, primarily manifesting as nonlethal autophagy that promotes cell survival. To induce cell death through targeted intervention that triggers autophagy, the required level of autophagic flux must be significantly greater than both baseline and survival-promoting autophagic flux. The study of autophagy in tumors requires a combination of multiple models (Kong et al., 2023), including organoid (Wei et al., 2023), zebrafish (Zhang et al., 2023), nude mouse (Yang et al., 2023) and microtumor models (Li Y. et al., 2024), to provide standardized experimental platforms for disease genesis and therapeutic mechanisms to help validate the efficacy of potential therapeutic targets and to further investigate the clinical candidates that trigger autophagy drug efficacy and pharmacological safety issues.

### 5.2 Potential of autophagy inhibitors in tumor angiogenesis therapy

**FIGURE 4:**
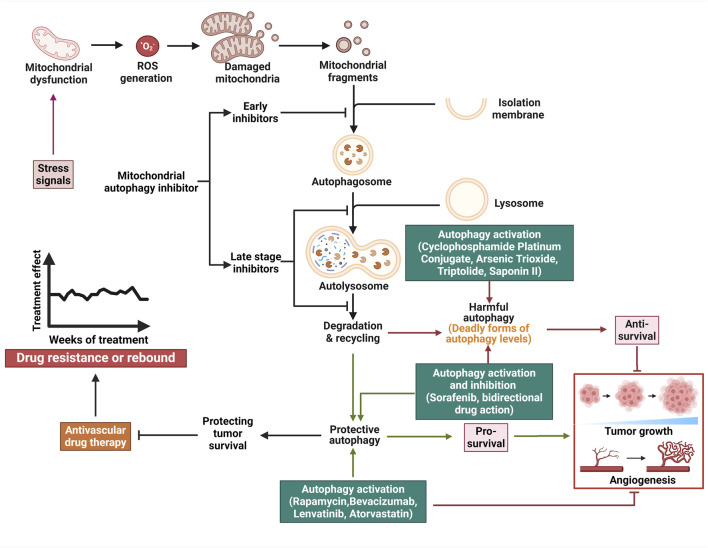
Role of autophagy in patients whose tumors escape antiangiogenic therapy. Interference with autophagy inhibition can lead to dysfunctional autophagy through interference with the early or late stages of the autophagy process. Notably, the drug-mediated activation of autophagy may trigger two different effects: on the one hand, excessive harmful autophagy may cause cellular damage; on the other hand, protective autophagy may play a critical cytoprotective role. The process of tumor evasion by antiangiogenic therapy may ultimately lead to the development of drug resistance or disease recovery through the maintenance of tumor cell survival and proliferation. Therefore, a combination therapeutic strategy using autophagy inhibitors against drug-induced protective autophagy may become an effective means to overcome drug resistance. This combined therapeutic strategy is expected to significantly improve the efficacy of antiangiogenic therapy by blocking the protective autophagy pathway.

In addition to the surgical removal of tumors, most tumors still require systemic treatment with oral medications, such as propranolol, for the treatment of infantile hemangiomas (Qiu et al., 2021; Yang et al., 2019). Pharmacological inhibitors of autophagy are usually categorized into early inhibitors, which inhibit the nucleation, extension and maturation stages of autophagosomes (excluding fusion with lysosomes and cargo degradation), and late inhibitors, which inhibit the lysosomal degradation of autophagosomes and their cargo (Chandra et al., 2020). A related microtumor angiogenesis microarray revealed that autophagy is highly activated near the endpoints of angiogenic sprouts and that autophagy inhibition leads to severe defects in angiogenic sprouts (Lee et al., 2024). Most studies have shown that tumor-protective autophagy triggered by antitumor drugs can be inhibited by the addition of autophagy inhibitors (Figure 4), enhancing the antitumor effects of these drugs (Liu et al., 2019). For example, bevacizumab inhibits angiogenesis and tumor xenograft growth while activating protective autophagy in the treatment of hepatocellular carcinoma, and the combination of autophagy inhibitors can further inhibit tumor growth (Guo et al., 2012). With respect to the therapeutic effects of levatinib (Xue et al., 2023) and atorvastatin (Yang et al., 2010) on tumors, the inhibition of autophagy likewise enhances drug cytotoxicity, apoptosis, and antiangiogenic ability, among other effects. Different tumor samples exhibit unique autophagy profiles and typically display enhanced autophagy activity compared with surrounding healthy tissues. Compared with tumors with high autophagy activity, tumors with low autophagy activity are often more sensitive to various chemotherapeutic agents and radiation therapy (Levy et al., 2014; Yamazaki et al., 2020). By culturing tumor organoids, specific drug regimens that effectively induce cell death can be identified in advance (Weeber et al., 2017). In most cases, autophagy protects tumor cells; therefore, inhibiting autophagy may be a superior strategy when antiangiogenic therapies are used to enhance antiangiogenic effects. Although interventions targeting autophagy hold promise as a viable approach to regulating cell death for tumor therapy, accurate quantification of autophagic flux is critical for successful clinical translation. This requires a comprehensive assessment of autophagy flux-related parameters, such as the baseline autophagosome turnover rate of the tumor itself.

## 6 Conclusion

In this work, we discuss the close association between autophagy and tumor angiogenesis in detail, aiming to explore the dual role of angiogenesis in tumor development and to elucidate the potential clinical applications of autophagy activators and inhibitors in antiangiogenic therapy, providing a solid theoretical foundation for understanding the new mechanisms of antitumor therapy. Recent studies on autophagy have focused on a single function (protective, lethal, or mediating a single mode of death). However, both autophagy itself and its regulation of cell death are dynamic processes. For example, a simple increase in the number of autophagosomes does not necessarily imply an increase in autophagy but may imply that autophagy stagnates at a late stage, which distinguishes between two autophagic flux scenarios: increased autophagy turn-on (increased autophagic flux with no damage to the single autophagic cycle) and decreased autophagy turn-off (impaired autophagosome clearance and defective catabolic function) (Levine and Kroemer, 2008). Currently, no highly targeted autophagy inhibitors or activators are available for use in humans in clinical practice, and the molecules currently used for this purpose (such as chloroquine and hydroxychloroquine) have various off-target effects with other therapeutic implications. The true impact of autophagy on tumor progression may be context dependent. Before autophagy-related functions are applied in clinical practice, clarifying the specific characteristics of a tumor’s dependence on autophagy, including its promoting or inhibitory effects, as well as the corresponding cell and tissue specificity, is essential. Furthermore, the high heterogeneity of the tumor microenvironment is likely to result in inconsistent therapeutic outcomes with this approach. All autophagy activators and inhibitors are currently in the preclinical exploration phase; their specific clinical application still requires extensive preclinical research and clinical validation to ensure the safety and efficacy of these drugs. Further attention needs to be given to the flexibility of the cellular role of autophagy, the complete changes in autophagy should be dynamically investigated, the potential mechanisms of autophagic conversion in tumors should be explored, and the exploration of clinically applicable target drugs, autophagic pathways and autophagy-sensitive indices should be combined to translate research related to autophagy and apply it in clinical treatments.
